# General practitioners’ and psychiatrists’ attitudes towards antidepressant withdrawal

**DOI:** 10.1192/bjo.2020.48

**Published:** 2020-06-18

**Authors:** Joanne McCabe, Mike Wilcock, Kate Atkinson, Richard Laugharne, Rohit Shankar

**Affiliations:** University of Exeter Medical School Knowledge Spa, Royal Cornwall Hospital, Truro, UK; Royal Cornwall Hospitals NHS Trust, Treliske, Truro, UK; Cornwall Partnership NHS Foundation Trust, Truro, UK; University of Exeter Medical School Knowledge Spa, Royal Cornwall Hospital, Truro; and Caradon CMHT, Trevillis House, Lodge Hill, Cornwall Partnership NHS Foundation Trust, Truro, UK; University of Exeter Medical School Knowledge Spa, Royal Cornwall Hospital, Truro; and Adult ID, Neurodevelopmental services Truro, Cornwall Partnership NHS Foundation Trust, Truro, UK

**Keywords:** Antidepressants, withdrawal side-effects, public mental health, public opinion

## Abstract

**Background:**

There has been a recent rise in antidepressant prescriptions. After the episode for which it was prescribed, the patient should ideally be supported in withdrawing the medication. There is increasing evidence for withdrawal symptoms (sometimes called discontinuation symptoms) occurring on ceasing treatment, sometimes having severe or prolonged effects.

**Aims:**

To identify and compare current knowledge, attitudes and practices of general practitioners (GPs) and psychiatrists in Cornwall, UK, concerning antidepressant withdrawal symptoms.

**Method:**

Questions about withdrawal symptoms and management were asked of GPs and psychiatrists in a multiple-choice cross-sectional study co-designed with a lived experience expert.

**Results:**

Psychiatrists thought that withdrawal symptoms were more severe than GPs did (*P* = 0.003); 53% (22/42) of GPs and 69% (18/26) of psychiatrists thought that withdrawal symptoms typically last between 1 and 4 weeks, although there was a wide range of answers given; 35% (9/26) of psychiatrists but no GPs identified a pharmacist as someone they may use to help manage antidepressant withdrawal. About three-quarters of respondents claimed they usually or always informed patients of potential withdrawal symptoms when they started a patient on antidepressants, but patient surveys say only 1% are warned.

**Conclusions:**

Psychiatrists and GPs need to effectively warn patients of potential withdrawal effects. Community pharmacists might be useful in supporting GP-managed antidepressant withdrawal. The wide variation in responses to most questions posed to participants reflects the variation in results of research on the topic. This highlights a need for more reproducible studies to be carried out on antidepressant withdrawal, which could inform future guidelines.

Antidepressant use has increased in recent years, with the number of prescriptions in England doubling in the past decade from the number in the mid-2000s.^1^ In 2018 the number of antidepressant prescriptions in England was 70.9 million.^2^ While antidepressants are generally considered beneficial to many of the people taking them, there are risks of withdrawal symptoms when patients later discontinue treatment. These are also known as discontinuation symptoms. A range of problems varying in intensity and duration have been reported, including increased anxiety, flu-like symptoms, insomnia, nausea, imbalance, sensory disturbances, hyperarousal, ‘brain zaps’, depersonalisation and sensory disturbances.^1,3,4^ The UK's National Institute for Health and Care Excellence (NICE) guidelines have been recently updated and state that ‘whilst the withdrawal symptoms which arise when stopping or reducing antidepressants can be mild and self-limiting, there is substantial variation in people's experience, with symptoms lasting much longer (sometimes months or more) and being more severe for some patients’.^5^ A recent all-party parliamentary group concluded that ‘It is incorrect to view antidepressant withdrawal as largely mild, self-limiting and of short duration’.^6^ This evidence suggests that withdrawal symptoms could be worse than those identified by NICE. The Royal College of Psychiatrists says that the potential benefits and harms of antidepressants, including withdrawal, should be discussed with the patient.^7^ The exact pathophysiology of withdrawal symptoms is unclear, but previous studies have hypothesised that they are a response to long-term physiological adaption of cerebral neural systems, or that they could be caused by a rapid decrease in serotonin availability when the treatment ends abruptly.^3^

## Issues with antidepressant withdrawal symptoms

There are various confounders surrounding the issue of withdrawal symptom reporting. For example, withdrawal symptoms can be mistaken for relapse, prompting re-starting of the antidepressant or changing to an alternative one, extending antidepressant use.^1^ In terms of patient perspective, fewer than 2% of antidepressant users recall being told by the prescriber about any withdrawal effects, or potential difficulties coming off the drugs.^1^ Patients who reported lack of knowledge of withdrawal symptoms also experienced greater adverse effects during withdrawal.^3^ Another poorly researched concept is the ‘nocebo’ effect – the expectation of feeling worse on discontinuation.^1^ Withdrawal effects can vary depending on the drug. For example, they may occur less frequently and less severely with longer-acting agents such as fluoxetine,^3,8^ giving rise to the option of switching to fluoxetine before stopping antidepressants completely, to potentially reduce withdrawal effects.^8^ Generally, selective serotonin reuptake inhibitors (SSRIs) with a shorter half-life seem to have worse withdrawal effects.^3^ For example, paroxetine has been found to have a higher incidence of antidepressant withdrawal when compared with fluoxetine and sertraline.^8,9^ It is not known exactly why this is the case but withdrawal severity is believed to depend on the elimination half-life of the drug and the patient's rate of metabolism.^3^

## Alleviating antidepressant withdrawal symptoms

Approaches have been suggested to reduce withdrawal symptoms, but there is conflicting evidence. One example is using cognitive–behavioural therapy (CBT) during withdrawal to promote understanding that any symptoms are temporary and due to withdrawal, rather than indicating an inability to cope without the medication.^3^ Patients who experienced worse adverse symptoms when commencing antidepressant treatment were more likely to suffer withdrawal symptoms; therefore, identifying these people could allow closer monitoring during withdrawal.^10^

Overall, literature about antidepressant withdrawal seems to differ from the NICE guidelines particularly in that there could be more advice about alleviating symptoms. There is little research exploring what professionals do in practice to inform and manage withdrawal symptoms, which in turn can influence patient outcomes.

To our knowledge, no study has systematically examined and compared how different groups of prescribers perceive withdrawal effects of antidepressants. Knowledge and understanding of general practitioners’ (GPs’) and psychiatrists’ attitudes towards withdrawal symptoms may allow identification of gaps in guidelines, policy or training.

## Aims

This study aimed to identify current knowledge, attitudes and practices of GPs and psychiatrists in Cornwall, UK (population: 538 000) concerning antidepressant withdrawal symptoms.

## Method

We completed a cross-sectional study of the opinions and perceptions of GPs and psychiatrists. The two groups were asked to complete a very similar questionnaire, with the GP survey consisting of nine questions and the psychiatrists’ survey having 14 questions (Supplementary information 1 and 2, available at https://doi.org/10.1192/bjo.2020.48). The questionnaires contained questions assessing perceptions about, and approach to, antidepressant withdrawal. Both surveys consisted of a mix of questions with predetermined answers, questions requiring the answer to be entered, and one question that allowed for free-text comments. The questionnaires were constructed by the authors on the basis of a review of the literature and were co-designed with a lived experience expert. Some limited demographic details were collected from the psychiatrists, although for both groups the survey was anonymous. The method of delivery of the questionnaire differed for the two groups. An edited summary of the questions is included in the Appendix.

### GPs

Across Cornwall, locality-based prescribing meetings are held in the North, Central and West GP localities of NHS Kernow, the clinical commissioning group (CCG) for the county, four times a year. These 12 meetings a year, organised by the CCG's Medicines Optimisation Team, are intended to have a focus on clinical prescribing and medicines optimisation. A GP prescribing lead from each primary care practice of the county is invited to attend these meetings and disseminate the learning to other GPs within their own practice. The questionnaire was handed out to GPs attending the winter 2019 meetings.

### Psychiatrists

For the psychiatrists, an introductory email and electronic link to the survey were emailed out to all 60 practitioners employed by Cornwall Partnership NHS Foundation Trust purposively selected through established contacts. A reminder email was sent 1 week later. Ten of these were psychiatric trainees (core training years CT1–3) and 50 were in senior posts (registrars in specialty training years ST4–6, consultants and associate specialists). For simplicity, this paper will refer to this group as ‘psychiatrists’ and the group from GP meetings as ‘GPs’.

Analysis of data was performed using Microsoft Excel (365 version, 2004). Descriptive statistics were used to describe and summarise the data highlighting the main elements of the study. Mann–Whitney U-tests were also used to evaluate whether there was any difference in responses between the two groups. IBM SPSS Statistics 26 for Windows was used to carry out these statistical tests.

### Ethics and participation consent

No ethical permission was required as this was a study to evaluate knowledge and attitudes as part of a service evaluation. Furthermore, it involved a group of medical practitioners where consent was implicit by participation. All participants were advised at the start of the study that participation was voluntary and their replies, i.e. data, would be anonymised and analysed. We also used the NHS Research Authority tool (http://www.hra-decisiontools.org.uk/research/index.html), which helped confirm that no ethics approval was needed for this project (Supplementary information 3).

## Results

There are 60 GP surgeries (primary care centres) within NHS Kernow CCG. The three meetings in winter 2019 were attended by a total of 53 GPs (88% attendance), with completed questionnaires returned by 42 (79%) of the attendees. No other GP characteristics were recorded. Of the 60 doctors and associate specialists working for psychiatry services in Cornwall Partnership NHS Foundation Trust, 26 responded to the electronic survey (43% response rate): consultants (*n* = 17); associate specialists (*n* = 2); speciality doctor (*n* = 1); ST4–6 trainee (*n* = 1); CT1–3 trainees (*n* = 4); 1 respondent did not state their level of experience so it is unknown. A range of specialties were represented: general adult psychiatry (*n* = 12); child and adolescent psychiatry (*n* = 7); complex care and dementia psychiatry (*n* = 4); psychiatry of intellectual disability (*n* = 2); 1 respondent did not give their specialty.

For the purposes of this paper, we will focus on the answers to the questions concerning duration of withdrawal symptoms, severity of withdrawal symptoms, their frequency of occurrence, what might prompt stopping antidepressants, what proportion of patients approached the respondents about stopping their antidepressants, how respondents would withdraw them, who they might work with and how often they discuss potential withdrawal symptoms before commencing treatment.

When asked how long they believe withdrawal symptoms typically last for, 53% (22/42) of the GPs and 69% (18/26) of the psychiatrists perceived the duration to be between 1 and 4 weeks (Table 1). There was no statistical difference between the two groups (*P* = 0.979).

**Table 1:** Perception of the typical duration of withdrawal symptoms, as reported by general practitioners (*n* = 42) and psychiatrists (*n* = 26)

Typical duration of withdrawal symptoms	General practitioners, *n* (%)	Psychiatrists, *n* (%)
No withdrawal symptoms	0 (0%)	0 (0%)
1–3 days	2 (5%)	1 (4%)
4–6 days	4 (10%)	1 (4%)
1–2 weeks	10 (24%)	7 (27%)
2–4 weeks	12 (29%)	11 (42%)
1–2 months	9 (21%)	3 (12%)
2–4 months	4 (10%)	1 (4%)
4–6 months	2 (5%)	1 (4%)
Over 6 months	0 (0%)	1 (4%)

**Fig. 1:**
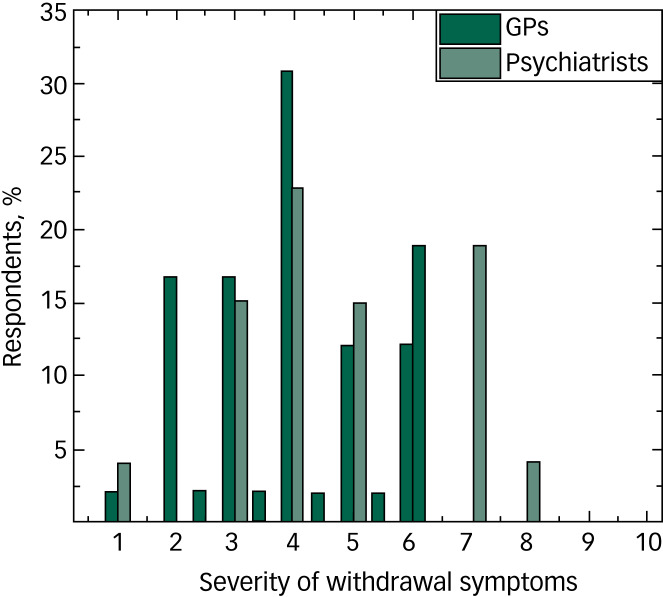
Perceived severity of withdrawal symptoms, as reported by general practitioners (GPs) (*n* = 42) and psychiatrists (*n* = 26).

As regards the severity of withdrawal symptoms, on average (on a scale of 1 to 10, where 1 is no negative effect at all and 10 is life-threatening) the mean value for the GPs’ responses was 3.8 (s.d. = 1.3), although the range was 1–6. The mean value for psychiatrists was 5 (s.d. = 1.7) and the range was 1–8. On the e-questionnaire completed by the psychiatrists, they were only able to select whole-number answers. On the paper survey that was completed by the GPs, some chose to give ranges. For the purposes of analysis, the midpoint of the range they gave was used. The results for this question can be seen in Fig. 1. There is a statistical difference between the two groups for this question (*P* = 0.003), with the psychiatrists perceiving more severe withdrawal symptoms than the GPs.

The proportion of people believed to be affected by withdrawal symptoms when stopping antidepressants is shown in Table 2, with just under half of our GP respondents (45%) and just under one-third of the psychiatrists (31%) perceiving that 21–40% of patients are affected when withdrawing antidepressants. There is no statistical difference between the two groups (*P* = 0.606).

**Table 2:** Perceived proportion of patients affected by withdrawal symptoms, as reported by general practitioners (*n* = 42) and psychiatrists (*n* = 26)

Proportion affected	General practitioners, *n* (%)	Psychiatrists, *n* (%)
0%	0 (0%)	0 (0%)
1–20%	9 (21%)	6 (23%)
21–40%	19 (45%)	8 (31%)
41–60%	8 (19%)	8 (31%)
61–80%	5 (12%)	4 (15%)
81–100%	1 (2%)	0 (0%)

When asked what might prompt them to discuss stopping antidepressants with a patient (such as any particular indications or time periods), five main themes emerged for each group, as shown in Table 3.

**Table 3:** Main five reasons mentioned for ceasing treatment, as reported by general practitioners (*n* = 38) and psychiatrists (*n* = 26)

Reasons	General practitioners, *n*	Psychiatrists, *n*
Duration of treatment	26	11
Patient request	9	10
Side-effects	8	21
Routine medication review	8	0
Improvement in patient symptoms	7	8
Lack of efficacy	0	13

All but four of the GPs provided free-text answers to a question about the three main concerns that might prompt them to discuss with a patient whether to stop antidepressants (such as any particular indications or time periods). The main headings are as follows: duration of treatment, for example after 6 months or after 1 year, was mentioned by 26 GPs; 9 GPs said that the patient asking to end treatment might act as a prompt; treatment side-effects and a routine medication review were each mentioned by 8 GPs; and noting an improvement in the patient's symptoms was cited by 7 GPs.

All the psychiatrists provided free-text answers to this question. The main headings are as follows: side-effects (21 responses); lack of efficacy (13 responses); duration of treatment (11 responses); prompted by the patient asking to end treatment (10 responses); noting an improvement in the patient's symptoms (8 responses).

When the GPs were asked to estimate what proportion of their patients who are on antidepressants approach them about stopping their antidepressant medication, the mean result was 20%, although the range was 2–100%. The mean result from psychiatrists was 23% (range 0–100%), from the 24 out of 26 respondents who gave a percentage.

The question on how they would typically go about withdrawing a patient from antidepressants elicited the responses in Table 4 (respondents could tick all that applied).

**Table 4:** Methods used for withdrawing a patient from antidepressants, as reported by general practitioners (*n* = 42) and psychiatrists (*n* = 26)

Pre-determined answer	General practitioners, *n* (%)	Psychiatrists, *n* (%)
Taper with reduced solid dose formulation (tablets/capsules) doses	39 (93%)	25 (96%)
Taper with liquid form	9 (21%)	5 (19%)
Complete immediate withdrawal	6 (14%)	0 (0%)
Switch to another antidepressant drug first	1 (2%)	3 (12%)
Use another drug to alleviate withdrawal symptoms	0 (0%)	5 (19%)
Other (please state)	0 (0%)	1 (4%)

The psychiatrist who responded ‘other’ stated that they would ‘Check the Maudsley guidance and follow that’.

When asked who they might consult/work with to support a patient through withdrawal and staying off medication (this could include professionals and non-professionals), the most common answers from the GPs were: social prescriber, mentioned by 12 GPs; no other support needed (or available), mentioned by 10; and consultant psychiatrist or other mental health counsellor, each mentioned by 7. The main responses from the psychiatrists were: patient/carer/family, mentioned by 13; GP, by 10; pharmacist or care coordinator, each mentioned by 9; and other counsellor/mental health practitioner, mentioned by 5.

As regards how often there is a discussion of potential withdrawal symptoms with the patient before commencing antidepressant treatment (Table 5), there was no statistical difference between the two groups (*P* = 0.438).

**Table 5:** How often general practitioners (*n* = 42) and psychiatrists (*n* = 26) said there was a discussion of potential withdrawal symptoms with the patient before commencing antidepressants

Pre-determined answer	General practitioners, *n* (%)	Psychiatrists, *n* (%)
Always	9 (21%)	10 (38%)
Usually	24 (57%)	9 (35%)
Sometimes	3 (7%)	4 (15%)
Rarely	6 (14%)	3 (12%)
Never	0 (0%)	0 (0%)

The final question allowed for free-text comments on antidepressant withdrawal, to which nine GPs and ten psychiatrists responded. Three GPs perceived that severe withdrawal was not a problem in their experience (these GPs scored the severity of withdrawal symptoms as 3, 4 and 5). Two other GPs noted that patients often stop taking antidepressants when they feel better, often without consulting their doctor.

## Discussion

This small study looked to understand a number of issues related to the withdrawal of antidepressants against a background of controversy and fierce debate on the benefits and harms of this class of medicine.^11^ It is a study that involved a full ecosystem of mental health management, i.e. primary and secondary care across a single CCG and geographical area (Cornwall) that covers about 1% of the UK population. We found some differences and similarities in views both within the two groups of healthcare professionals and also across the two groups.

### Limitations

The relatively small sample (42 GP responses and 26 psychiatry responses) means that there is a risk of type 2 error, and genuine differences might be revealed by a larger sample. To address this limitation, we hope to extend this study to the rest of the South West of the UK. The current study has been a useful pilot.

We recognise the limitations with this small study undertaken in just one geographical region and, for the GPs, with a self-selected group of healthcare professionals. Cornwall is a predominantly rural and White (99.5%). It is also one of the most deprived areas not only of the UK but also of the European Union. Thus, it is possible that healthcare professionals working in different healthcare systems may hold different views on this subject. Further studies to cover wider areas of the UK would show whether these results are reproducible.

We also used a survey that could have introduced biases such as recall bias and answering tendencies. Triangulation with objective assessments of patient management such as case-note audits would have added to our characterisation of current practice.

Our questions were about withdrawal of antidepressants for any indication and respondents may actually have different views on severity or absence of withdrawal symptoms depending on the condition being treated.

### Main findings

A systematic review reported that, overall, seven out of ten studies found that a large proportion of participants reported experiencing antidepressant withdrawal symptoms for more than 2 weeks.^1^ This is somewhat contrary to our findings. The majority of respondents from both groups of our study thought that withdrawal symptoms typically last between 1 and 4 weeks: 53% (22/42) of GPs and 69% (18/26) of psychiatrists. However, a wide range of answers was given. For example, the GPs perceived that withdrawal symptoms could last typically for anywhere between a matter of days (6 respondents (15%) suggested less than a week) to 4–6 months (2 respondents (5%)), which was similar to the perceptions of some of the psychiatrists. Only 14 (33%) of our GPs and 12 (46%) of our psychiatrists considered that the proportion of people affected by withdrawal symptoms is greater than 40%.

#### Incidence and severity of withdrawal symptoms

As well as the heated debate about the merits of antidepressants, it is argued that another important discrepancy between the scientific literature and prevailing beliefs held by leading psychiatrists concerns withdrawal symptoms on discontinuation of antidepressant medication.^12^ According to several studies, severe and persistent withdrawal reactions affect up to 50% of antidepressant users. Others quote that more than 50% of people who attempted to stop antidepressants experienced withdrawal effects and nearly 50% of those experiencing withdrawal effects described them as severe.^1^ Notably, in our study the psychiatrists considered withdrawal effects to be more severe than the GPs. It is possible that this is due to differences in patient populations encountered by the two groups, with psychiatrists perhaps more likely than GPs to encounter patients who have been on antidepressants for longer periods of time, which could make withdrawal symptoms worse. Also, relationships have been shown between mental disorders and medical comorbidities,^13^ so it is possible that those with more severe mental disorders or more complex needs (who are therefore more likely to need psychiatrist input) could show more severe withdrawal symptoms owing to an interplay with other medical needs.

#### Prescribing practices

In an analysis of 52 recorded primary care consultations for depression, anxiety and stress, patients resisted treatment because of doubts about its efficacy based on previous experiences, fears about dependency and/or side-effects, and concerns about attending group therapy.^14^ Historically, it was known than many patients on longer-term courses of antidepressants were not being appropriately reviewed.^15^ A study of Scottish GPs during 2014–2015 noted that the lack of proactive medication reviews (e.g. patients only present in crisis) contributed to further antidepressant prescribing growth over time.^16^ Twenty-six (62%) of our GPs and 11 (42%) of our psychiatrists indicated that duration of treatment would make them consider withdrawal of treatment.

#### Patient support during withdrawal

It is interesting that 12 GPs (29%) identified a social prescriber as someone with whom they would consult/work to support a patient through withdrawal and staying off medication, yet the evidence base for social prescribing is very limited. It is recognised that there is a need to consider support for health professionals in the management of antidepressant medication and discussions of discontinuation in particular.^17^

Nine (35%) psychiatrists said they would involve pharmacists and have access to them, but GPs did not mention seeking help from pharmacists. This could raise the suggestion of whether it would be valuable to encourage involvement of community pharmacists in antidepressant withdrawal that is managed by GPs. A recent study showed the potential support that community pharmacists could give to patients on antidepressants, such as by monitoring adherence and efficacy of treatment.^18^

#### Patient advice on potential withdrawal symptoms

Approximately three-quarters of respondents claimed that they always or usually provide information to patients about potential antidepressant withdrawal symptoms before commencing them. This is in sharp contrast to an online survey of a self-selected convenience sample of patients, mainly from Europe, North America and Australasia, in which less than 1% replied that had been told anything about withdrawal effects.^19^

### Implications for the patient

Our co-author, K.A., a lived experience expert who helped design the study and questionnaires, shares her perspective in response to the results of this study:

‘This project highlights that there still remain gaps between perceptions of clinicians to those of patients. It is my personal experience and those who I represent that withdrawing from antidepressants would be much longer lasting and have more serious withdrawal effects than what has been reported clinically here. Personally, and I expect for many, it is a scary project to undertake. There is a lack of resources and support available to support person-centred withdrawal. I don't recall ever being warned by anyone treating me about withdrawal effects beyond the generic message of don't stop suddenly. The study highlights that many of the concerns I have outlined have not been objectively examined.’

### Implications for practice

One of the key findings of this study is that roughly 75% of psychiatrists and GPs say they always or usually warn patients about withdrawal side-effects, but patient surveys say that only 1% are warned. This could be because clinicians are rapidly changing as awareness is growing, which would be positive, or because there is a communication gap and the message is not clear enough. The mismatch between the findings of our study and previous patient surveys could also mean that some patients who are told about withdrawal symptoms simply do not recall this. Both of possible negative explanations suggest a need to encourage more GPs and psychiatrists to inform patients of potential withdrawal effects and to do this in a way that patients will remember.

### Implications for policy

The results highlight that a proportion of psychiatrists involve pharmacists when withdrawing a patient from antidepressants, but GPs do not. Encouraging involvement of community pharmacists to support GPs in doing this may be a useful source of support.

### Implications for research

Antidepressant withdrawal is a serious public health concern that warrants more research and adequate appraisal by academic psychiatry.^12^ It is interesting to note such a high variation in responses from all participants. The diversity of responses reflects the wide variation in results from research studies. This shows a need for more reproducible studies to be carried out into the patient experience of antidepressant withdrawal. We suggest that future studies exploring this topic should carefully consider their methodology to make it as representative as possible. This means that a more precise impression could be given of the proportion of patients who are affected by antidepressant withdrawal, how often the symptoms typically last and so on. This could also include, for example, identifying patient groups who are more at risk of severe withdrawal symptoms. This could inform future guidelines to make them more specific, so that GPs and psychiatrists are able to provide more consistent evidence-based advice and management to patients, which could improve patient outcomes.

## Data Availability

The data that support the findings of this study are available from the corresponding author on reasonable request.

## Supplementary material

For supplementary material accompanying this paper visit http://dx.doi.org/10.1192/bjo.2020.48. click here to view supplementary material

## Appendix

Edited summary of topics covered by the questions asked in the study:

- duration of withdrawal symptoms
- severity of withdrawal symptoms
- proportion of people affected by withdrawal symptoms when stopping antidepressants
- prompts for stopping antidepressants
- proportion of patients who approach the respondent about stopping antidepressants
- how antidepressants are typically withdrawn
- who might be consulted/worked with to support a patient to withdraw and stay off antidepressants
- frequency of discussion of withdrawal symptoms with patients before commencing antidepressant treatment
