# Supplementary material for: General practitioners’ and psychiatrists’ attitudes towards antidepressant withdrawal
Source: BJPsych Open. 2020 Jun 18;6(4):e64. doi: 10.1192/bjo.2020.48 (PMC7345735; doi:10.1192/bjo.2020.48)
Supplement: Supplementary file 1 [file bjosup.zip › S2056472420000484sup002.zip!/Supplementary information 1 - GP survey tool .pdf]

A survey of GP attitudes to antidepressant withdrawal

You may be aware that there is increasing research being carried out concerning the duration and severity of withdrawal symptoms from stopping antidepressant medication. This brief survey aims to gain an understanding of your views and how you approach this topic in practice. Please feel free to write any extra comments at the end.

1. How long do you believe withdrawal symptoms typically last for? (please tick one response)

- There are none
- 1-3 days
- 4-6 days
- 1-2 weeks
- 2-4 weeks
- 1-2 months
- 2-4 months
- 4-6 months
- Over 6 months

2. How severe do you believe withdrawal symptoms are, on average? (on a scale of 1-10, where 0 is no negative effect at all and 10 is life threatening)

Please state a number from 1 to10….

3. What proportion of people do you believe are affected by withdrawal symptoms when stopping antidepressants? (please tick one response)

- 0%
- 1-20%
- 21-40%
- 41-60%
- 61-80%
- 81-100%

4. What might prompt you to discuss with a patient about stopping antidepressant? (eg any particular indications, time periods etc.)

5. What proportion of your patients who are on antidepressants would you estimate have approached you about stopping their antidepressant medication?

Please write an approximate percentage……………………………

6. How would you typically go about withdrawing a patient from antidepressants? (please tick all that apply)

- Complete immediate withdrawal
- Taper with liquid form
- Taper with reduced solid dose formulation (tabs / caps) doses
- Switch to another antidepressant drug first (please specify which drug)

  ....…………………………………………………………………………..
- Use another drug to alleviate withdrawal symptoms (please specify which drug)

  ……………………………………………………………………………………
- Other (please state)

  …………………………………………………………………………………

7. Who might you consult/work with to support a patient through withdrawal and staying off medication? (including professional and non-professional roles)

....

……………………………………………………………………………………………………

....

8. How often do you discuss potential withdrawal symptoms with your patients prior to commencing antidepressant treatment? (please tick one response

- Always
- Usually
- Sometimes
- Rarely
- Never

9. Any brief written comments on this subject?....

Thank you for your time.
