# Supplementary material for: General practitioners’ and psychiatrists’ attitudes towards antidepressant withdrawal
Source: BJPsych Open. 2020 Jun 18;6(4):e64. doi: 10.1192/bjo.2020.48 (PMC7345735; doi:10.1192/bjo.2020.48)
Supplement: Supplementary file 1 [file bjosup.zip › S2056472420000484sup002.zip!/Supplementary information 2 - Psychiatrists survey tool.pdf]

A survey of psychiatrists' attitudes to antidepressant withdrawal

You may be aware that there is increasing research being carried out concerning the duration and severity of withdrawal symptoms from stopping antidepressant medication. This brief survey aims to gain an understanding of your views and how you approach this topic in practice. This includes all uses of antidepressant medication, not just for treatment of depression.

Please feel free to write any extra comments at the end.

1. What is your level of experience

Mark only one oval.

- CT1-3 Trainee
- ST4-6 Trainee
- Consultant for 0-10 years
- Consultant for over 10 years
- Other: ____________________

2. What is your specialty?

Mark only one oval.

- General adult psychiatry
- Child and adolescent psychiatry
- Old age psychiatry
- Other: ____________________

3. How long do you believe withdrawal symptoms typically last for?

Mark only one oval.

- There are none
- 1-3 days
- 4-6 days
- 1-2 weeks
- 2-4 weeks
- 1-2 months
- 2-4 months
- 4-6 months
- Over 6 months

4. How severe do you believe withdrawal symptoms are, on average? (on a scale of 1-10, where 0 is no negative effect at all and 10 is life threatening)

Mark only one oval.

	0	1	2	3	4	5	6	7	8	9	10	
0												10

5. What proportion of people do you believe are affected by withdrawal symptoms when stopping antidepressants?

Mark only one oval.

- 0%
- 1-20%
- 21-40%
- 41-60%
- 61-80%
- 81-100%

6. How many patients would you estimate you start on antidepressants per month? DO include patients for whom you have recommended starting antidepressants, even if you were not the person who changed the prescription.

Mark only one oval.

- 0-10
- 11-20
- 21-30
- 31-40
- 41-50
- More than 50

7. How does your previous answer compare to the number of people you withdraw from antidepressants per month? DO NOT include those switching to a different antidepressant without the intention of withdrawal.

Mark only one oval.

- Significantly more have started than withdrawn from antidepressants
- Slightly more have started than withdrawn from antidepressants
- About the same number have started as have withdrawn from antidepressants
- Slightly less have started than withdrawn from antidepressants
- Significantly less have started than withdrawn from antidepressants

8. What are the top 3 factors that might prompt you to discuss stopping antidepressants (eg any particular indications, time periods etc.)

9. What proportion of your patients who are on antidepressants do you estimate have approached you about stopping their medication? Please write an approximate percentage.

____

10. How would you typically go about withdrawing a patient from antidepressants? (please tick all that apply)

Check all that apply.

- [ ] Complete immediate withdrawal
- [ ] Taper with liquid form
- [ ] Taper with reduced tablet doses
- [ ] Switch to another antidepressant first
- [ ] Use another drug to alleviate withdrawal symptoms

Other: [ ] ________________________________

11. On the previous question, if you said you would switch to another antidepressant first, or use another drug to alleviate withdrawal symptoms, please specify which drug you would use.

12. Who might you consult/work with to support a patient throught withdrawal and staying off medication (including professional and non-professional roles)?

13. How often do you discuss potential withdrawal symptoms with your patients prior to commencing antidepressant treatment?

Mark only one oval.

- Always
- Often
- Sometimes
- Rarely
- Never

14. Any brief written comments on this subject?

This content is neither created nor endorsed by Google.

Google Forms
